# Transperineal 3D vector magnetic resonance elastography of the prostate is feasible in men with benign prostatic hyperplasia and lower urinary tract symptoms

**DOI:** 10.1007/s00261-026-05407-2

**Published:** 2026-04-08

**Authors:** Cody Johnson, Juan Pablo Gonzalez-Pereira, Matthew Grimes, Lu Mao, William Ricke, John Wei, Christopher Brace, Shane Wells

**Affiliations:** 1 University of Wisconsin–Madison, Madison, USA; 2 University of Michigan–Ann Arbor, Ann Arbor, USA

**Keywords:** Prostate, Magnetic resonance elastography, MRE, Benign prostatic hyperplasia, BPH, Lower urinary tract symptoms, LUTS

## Abstract

**Purpose:**

To establish feasibility of 3D vector magnetic resonance elastography of the prostate (pMRE) with a novel transperineal passive driver in men with benign prostatic hyperplasia/lower urinary tract symptoms (BPH/LUTS).

**Methods:**

Following informed consent, 21 men (median age: 41 years, IQR: 35–61) without (*n* = 6) or with mild (*n* = 9) and moderate/severe (*n* = 6) LUTS underwent multiparametric prostate MRI including pMRE, on a 3.0T MRI using a commercially available active driver and prototype transperineal passive driver. Prostate volume (PV), prostatic urethra length (PUL), postvoid residual (PVR) and bladder wall thickness (BWT) were measured. Regions-of-interest were drawn on the prostate TZ shear stiffness maps at the base and midgland. Mean TZ shear stiffness was calculated. The product of mean TZ shear stiffness and PUL [kPa x U length] were calculated. LUTS severity and uroflowmetry parameters, including peak (Qmax) and average (Qave) urine flow were measured with the International Prostate Symptom Score (IPSS) and a uroflowmeter, respectively. The Kruskal-Wallis rank sum test and Spearman correlation coefficients were used to assess relationships between imaging and clinical data. A p-value of < 0.05 was considered statistically significant.

**Results:**

Men with moderate/severe LUTS (median IPSS: 20, IQR: 13.25–23.25) were older (*p* = 0.008), had a higher BMI (*p* = 0.044), and a lower Qmax (*p* = 0.005) and Qave (*p* = 0.017) compared to men without and with mild LUTS. PV and PVR were similar across groups. Stiffness analysis included 19 men. Higher midgland TZ shear stiffness correlated with lower Qmax (ρ = -0.57; *p* = 0.017). Increase in [kPa x U length] correlated with a lower Qmax (ρ = -0.59; *p* = 0.015) and Qave (ρ = -0.51; *p* = 0.04). The correlation between Qmax, Qave and voided volume was higher with [kPa x U length] compared to mean TZ shear stiffness alone.

**Conclusions:**

pMRE with a novel transperineal passive driver is feasible and can be used to measure TZ shear stiffness. Higher TZ shear stiffness and longer PUL are associated with voiding dysfunction in men with moderate/severe LUTS.

## Introduction

Lower urinary tract symptoms (LUTS) include a spectrum of clinical symptoms associated with the storage and elimination of urine that can arise from neurogenic and non-neurogenic etiologies that affect the structure and/or function of the bladder, urethra/sphincter, and prostate. In men, LUTS are frequently attributed to benign prostatic hyperplasia (BPH), a histologic diagnosis that refers to the stromal and epithelial proliferation in the prostate transition zone (TZ) that often contributes to an increase in size of the gland. The enlarged prostate can lead to bladder outlet obstruction (BOO), bladder remodeling, and the emergence of lower urinary tract symptoms (LUTS) such as urgency, frequency and slow stream. LUTS are extremely common and increase in prevalence and severity with age. Importantly, similar patterns of LUTS may be attributed to non-BPH etiologies including overactive bladder (OAB), detrusor underactivity (DU), lifestyle choices, and medications [1–5]. This limits the accuracy and reliability of non-invasive tests that clinicians rely on to distinguish BOO from other causes of LUTS in ageing men, including, symptom scores often assessed with the International Prostate Symptom score (IPSS), prostate size, voiding function usually measured with uroflowmetry, and postvoid residual (PVR) [5–9]. Currently, the diagnosis of BOO requires multichannel urodynamics (UDS), an invasive study that is not commonly performed in clinical practice due to limited availability and lack of expertise. As a result, the vast majority of men with BPH/LUTS are treated for suspected but unconfirmed BOO based upon results from non-invasive tests. This contributes to substantial heterogeneity in patient reported outcomes and decreased quality of life [12–14].

Multiparametric MRI (mpMRI) has become the standard of care in the assessment of men with suspected prostate cancer, providing qualitative images of the prostate based upon T1- and T2-weighting for assessment by a radiologist. Magnetic resonance elastography (MRE), on the other hand, is a quantitative MRI technique used to measure the mechanical properties of a target organ. Tissue stiffness can be visualized and measured on a shear stiffness map (or ‘elastogram’), providing a noninvasive approach for diagnosing and monitoring diseases, often liver fibrosis in current clinical practice [15, 16]. Until recently, the lack of software and hardware solutions have limited the application of MRE to the prostate. Three-dimensional (3D) vector MRE, which images the wave field in all three spatial directions (*x*,* y*, and *z* axis) at every voxel, reduces directional bias from predecessor technology (2D MRE) thereby improving the accuracy of stiffness estimates, especially with complex tissue geometry and wave pathways, such as the pelvis [17]. Existing active-passive driver combinations utilizing a transabdominal approach required a low mechanical frequency to penetrate deep into the pelvis, resulting in poor image quality and low resolution elastograms. Further, this approach is limited by frequent artifacts associated with intervening anatomy including motion (bowel peristalsis), bowel gas, and urine in the bladder. We developed a novel passive driver and custom anchoring system that allows for a transperineal approach. This approach decreases the distance between the passive driver and prostate allowing for the application of higher mechanical frequencies and eliminates image-related artifacts associated with intervening anatomy from transabdominal drivers.

Therefore, the primary objective of this study is to demonstrate feasibility of 3D vector prostate MRE (pMRE) using a novel transperineal passive driver and organ-specific excitation frequency. The secondary objective is to explore the hypothesis that an increase in prostate TZ shear stiffness and urethral length, alter voiding dynamics and contribute to development of LUTS in men with BPH.

## Materials and methods

### Study design

This prospective, single-center, cross-sectional study was IRB-approved and HIPAA compliant. Enrollment began on January 1, 2023, and is ongoing. Participants included in this analysis were enrolled between January 1, 2023 and December 30, 2024. Informed consent was obtained from all participants. All participants attended a research clinic visit prior to magnetic resonance imaging (MRI), where a medical history and focused physical examination including age, vital signs, height, and weight were obtained by an experienced clinical investigator with access to the electronic medical record. A detailed medication history was obtained and documented. All participants completed the International Prostate Symptom Score (IPSS) questionnaire.

Participants were instructed not to void prior to the research clinic visit. At the beginning of the clinic visit, participants were provided standard instructions for voiding on a uroflowmeter (Laborie Flowmaster, Portsmouth, NH, USA). Uroflowmetry was obtained immediately before imaging, when participants experienced the urge to void.

The research clinic visit was followed immediately by a 50-min imaging session that included mpMRI and 3D vector pMRE. All participants completed a standardized tolerability questionnaire at the conclusion of the imaging session.

## Inclusion and exclusion criteria

Men 18 years and older, were recruited by a research coordinator from an existing volunteer database curated in the Department of Radiology or from Urology Clinic, at the University of Wisconsin, by a fellowship-trained (endourology) urologist, study team member and co-author (MDG). Those who completed the IPSS and signed written informed consent who did not meet any exclusion criteria were enrolled in the study. Exclusion criteria included: prior prostate treatments for cancer (surgery, radiation) or BPH/LUTS, use of medications or herbal supplements for BPH/LUTS within the last 6 months, metallic hip or pelvis implants, and/or contraindication(s) to MRI.

## Multiparametric MRI and 3D vector prostate MRE acquisitions

**Fig. 1:**
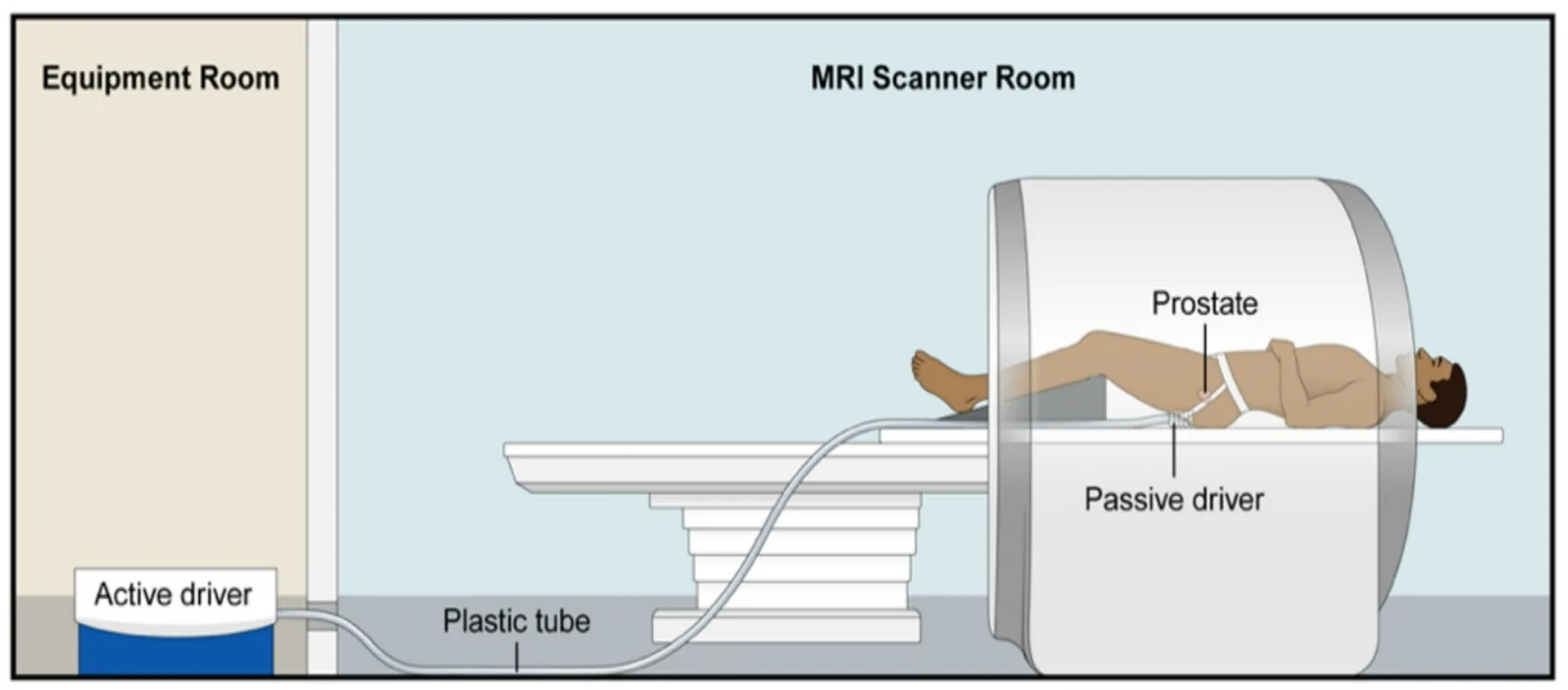
Magnetic resonance imaging (MRI) and 3D vector prostate magnetic resonance elastography (pMRE) setup in a 3.0T MRI suite (SIGNA Premier, GEHC, Waukesha, WI, USA)

Immediately following uroflowmetry, participants donned the custom anchoring device housing a 30 mL cylindrical passive driver fabricated from an FDA-compliant medical grade plastic resin (low-density polyethylene). Participants were then placed in a supine position on the MRI table, and a padded wedge was placed under the knees for comfort. The passive driver, centered on the perineum behind the scrotum and anterior to the rectum, was connected to the active driver (Resoundant Inc., Minneapolis, MN, USA), located outside of the scanner room, via plastic tubing (7.6 m long) (Fig. 1). Continuous acoustic harmonic waves were generated from the active driver across a frequency range of 80–110 Hz. The frequency of 90 Hz provided adequate wave propagation into the pelvis and image resolution of the prostate and was used for prostate TZ stiffness estimations for this study. pMRE, using a straight axial spin-echo echo-planar imaging (EPI) acquisition with 3D vector motion encoded gradients (MEG), and mpMRI, including axial T2-weighted (T2w) fast spin echo, diffusion-weighted imaging (DWI), apparent diffusion coefficient (ADC, *b*800 and *b*1500), and dynamic contrast enhancement (DCE), were performed at 3.0T (Premier, GE Healthcare, Waukesha, WI, USA) with a 30-channel phased array surface coil. pMRE pulse sequence parameters are provided in Table 1. Prior to the pMRE acquisitions, a sagittal T2-weighted (T2w) fast spin echo acquisition of the pelvis, covering the bladder and prostate, was obtained and the location and orientation of the passive driver was assessed and adjusted, as needed.

**Table 1:** 3D vector prostate magnetic resonance elastography (pMRE) pulse sequence parameters

Parameters	3.0T MRI (SIGNA Premier, GEHC)
Sequence	3D SE - EPI MRE
Plane	Axial
TR (ms)	1600
TE (ms)	~ 57.5
Bandwidth (kHz)	250
Field of view (mm)	260 × 260
Number of slices	30
Slice thickness (mm)	2.9
Gap between slices	0
Phase offsets	3
Fat Suppression	On
NEX	1
Acquisition matrix	Acquired 96 × 96, interpolated to 256 × 256
Acquired voxel size (mm)	1.7 × 1.7 × 2.9
Flip angle (°)	90
Typical scan time (sec)	90
MRE driver frequency (Hz)	90
MEG frequency (Hz)	70

*SE* Spin-echo, *EPI* Echo-planar imaging, *MRE* Magnetic resonance elastography,

*TR* Repetition time, *TE* Echo time, *NEX* Number of excitations,

*MEG* Motion-encoded gradients

pMRE data were processed using the Helmholtz equation to calculate the complex shear modulus (G*= G’ + iG”), where G* is the complex shear modulus, *G’* is the storage modulus, and *iG”* is the loss modulus [18]. The magnitude part of the shear modulus is henceforth referred to as shear stiffness [19]. The real and imaginary part of the shear modulus referred to as storage modulus and loss modulus, respectively, were not evaluated in this study.

## Data collection

### Clinical data

Participants age was recorded and body mass index (BMI) was calculated [BMI = weight (kg)/height^2^ (m)]. Participant LUTS severity was calculated as the sum of the IPSS from the questionnaire responses. Participants with a score of 0 were considered to have no LUTS. Participants with a score of 0–7, 8–19, and 20–35 were considered to have mild, moderate, or severe LUTS, respectively [20]. Uroflowmetry curves were assessed to ensure quality, and the following parameters were recorded: Qmax (mL/sec), Qave (mL/sec), voiding time (sec), and voided volume (mL).

### Multiparametric MRI data

Whole prostate and TZ volumes, bladder wall volume (BWV), and postvoid residual (PVR) were estimated from manual contours drawn on the axial and sagittal T2w images in Mimics (Materialize, version 21.0, Leuven, BEL) by a member of the investigative team with 5 years of experience, as previously described [21]. Mean bladder wall thickness was estimated from the bladder wall segmentation. Prostatic urethral length (PUL) was measured on the sagittal T2w image, from the bladder neck to the apex of the prostate.

## 3D vector prostate MRE data

A freehand, round region-of-interest (ROI) was drawn on the TZ of the shear stiffness map, at the level of the prostate base and midgland, referencing the axial T2w MRI to manually adjust to anatomic landmarks, on a DICOM viewer (Osirix MD, Geneva, CHE) by an abdominal radiologist and author (SAW) with 15 years of liver MRE and prostate MRI experience. The verumontanum was used as the anatomic landmark to identify the prostate midgland. The cranial most slice of the prostate that did not include the bladder neck was used as the reference for the prostate base. The ROIs were drawn to encompass the entire TZ while avoiding the edge of the prostate and obvious artifacts. Mean prostate TZ shear stiffness (kPa) from the TZ midgland and base was calculated. The product of mean TZ shear stiffness and PUL was recorded (kPa x U length).

### Statistical analysis

Continuous and categorical variables were summarized with frequency counts and percentages or median and interquartile range. The Kruskal-Wallis rank sum test was used to test the differences between groups of participants without LUTS, with mild and moderate/severe LUTS. Spearman correlation coefficients were calculated to assess the strength and direction of relationships between clinical data, mpMRI, and 3D vector pMRE. A p value < 0.05 was considered statistically significant. Multiple comparison correction was not performed in this exploratory study. All statistical analyses were performed using R version 4.2.2 (R Foundation for Statistical Computing, Vienna, AUT).

## Results

### Clinical data

Demographic and clinical characteristics are summarized in Table 2. Twenty-one men with a median age of 41 years (IQR: 35–61) and BMI of 28 kg/m^2^ (IQR: 24–31), respectively, were enrolled. The median IPSS for the entire cohort was 6.5 (IQR: 4–17.25.25). Six men had no LUTS (IPSS = 0), nine men had mild LUTS (median IPSS = 5, IQR: 3.5–6.5), and six men had moderate/severe LUTS (median IPSS = 20, IQR: 13.25–23.25). Men with moderate/severe LUTS were older (*p* = 0.008) and had a higher BMI (*p* = 0.044) than men without LUTS.

Uroflowmetry was not obtained in two participants, one with mild LUTS and one with moderate LUTS, thus excluded from voiding function analysis. Uroflow data was satisfactory for all other participants. Uroflowmetry data (19/21) are summarized in Table 2. Men with moderate/severe LUTS had a lower Qmax (12 vs. 32 mL/sec, *p* = 0.005) and Qave (5.8 vs. 15 mL/sec, *p* = 0.017) than men without LUTS. Voided volume, voiding time and PVR were similar across groups.

**Table 2:** Demographic, clinical, magnetic resonance imaging (MRI) and 3D vector prostate magnetic resonance elastography (pMRE)

Demographics (*n* = 21)	No LUTS	Mild LUTS	Moderate/Severe LUTS	*p*-value
	(*n* = 6)	(*n* = 9)	(*n* = 6)	
Age (years)	25 (23,36)	48 (36,61)	62 (55,65)	**0.008**
BMI (kg/m^2^)	27 (24,31)	24 (22,29)	34 (30,41)	**0.044**
Uroflowmetry (*n* = 19)	(*n* = 6)	(*n* = 8)	(*n* = 5)	
Qmax (mL/sec)	32 (28,39)	16 (10,21)	12 (10,16)	**0.005**
Qave (mL/sec)	15 (13.3,15.9)	8.0 (6.1,10.8)	5.8 (4.5,7.1)	**0.017**
Voided volume (mL)	287 (266,511)	219 (178,416)	167 (115,203)	*0.078*
Voiding time (sec)	28 (19,38)	31 (28,42)	31 (25,36)	> 0.9
Prostate and Bladder MRI (*n* = 21)	(*n* = 6)	(*n* = 9)	(*n* = 6)	
Postvoid residual (mL)	151 (59,256)	106 (29,254)	147 (58,162)	0.9
Prostate volume (mL)	21 (16,23)	27 (22,50)	36 (28,46)	0.2
Transition zone volume (mL)	7 (5,8)	11 (10,19)	17 (12,20)	**0.032**
Bladder wall thickness (cm)	0.3 (0.27,0.37)	0.32 (0.24,0.39)	0.27 (0.27,0.30)	> 0.9
Prostatic urethral length (PUL) (cm)	3.8 (3.6,4.1)	4.1 (3.8,4.4)	4.5 (4.4,4.8)	*0.072*
Prostate TZ Shear Stiffness (*n* = 19)	(*n* = 6)	(*n* = 8)	(*n* = 5)	
Base (kPa)	3.5 (3.4,3.6)	3.7 (3.5,4.5)	4.2 (3.9,4.2)	0.6
Midgland (kPa)	3.6 (3.5,3.6)	4.1 (3.7,4.7)	4.3 (4.0,4.4)	0.4
Mean (kPa)	3.6 (3.5,3.6)	3.9 (3.6,4.5)	4.3 (4.0,4.3)	0.5
kPa x U length (kPa·cm)	13.4 (12.6,13.9)	15.4 (13.7,19.9)	18.9 (15.2,20.6)	0.1

*LUTS* Lower urinary tract symptoms,* BMI* Body mass index,* Qmax * Peak flow, * Qave * Average flow,,*TZ * Transition zone,* kPa* kilopascals

### Imaging data

**Fig. 2:**
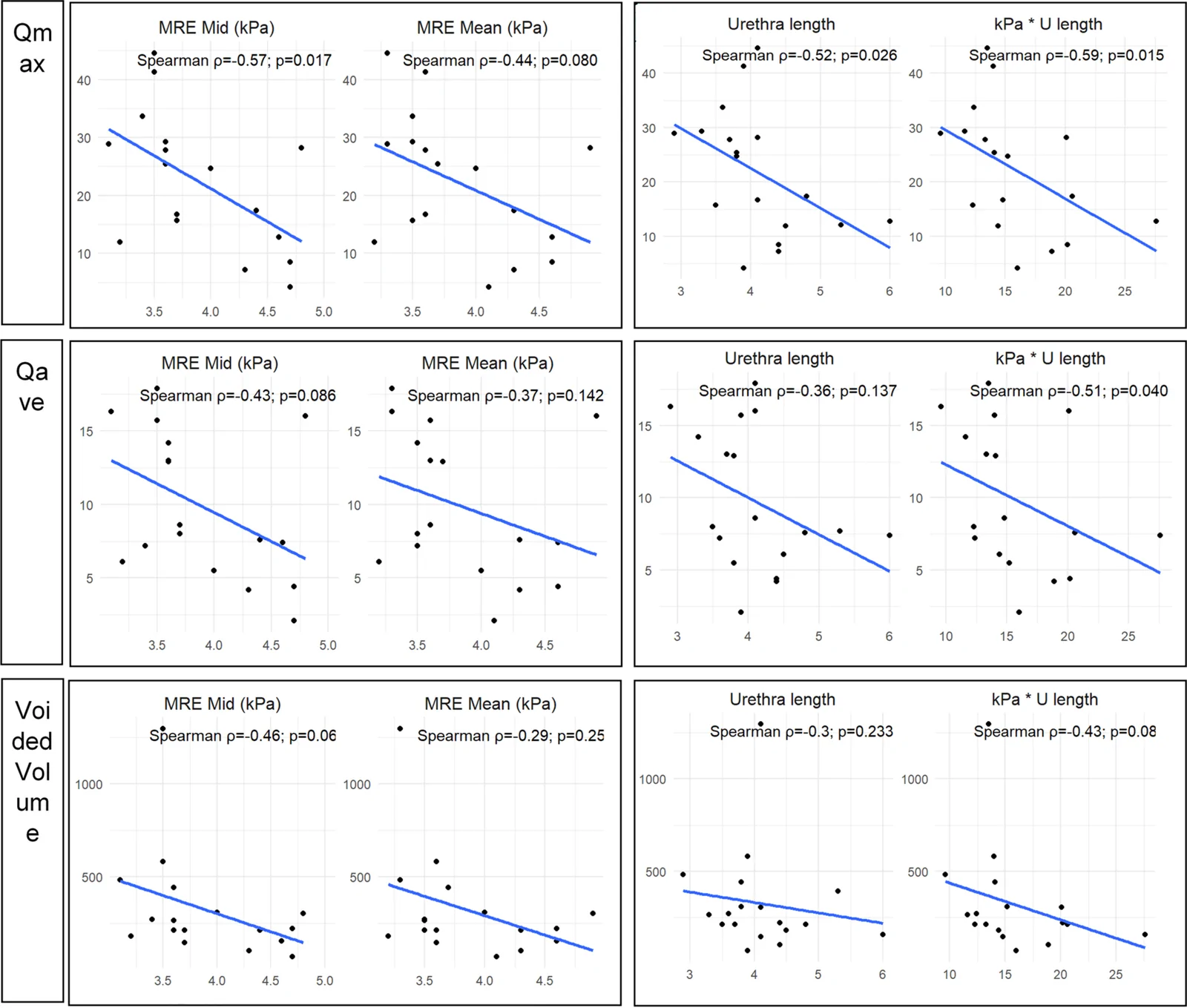
Spearman correlation coefficient graphs displaying the strength and direction of relationships between midgland (Mid) and mean prostate transition zone (TZ) shear stiffness in kilopascals (kPa), prostatic urethral length (U length), and the product of mean TZ shear stiffness and prostatic urethral length (kPa x U length) to uroflowmetry voiding parameters including maximum urine flow (Qmax), average urine flow (Qave) flow, and voided volume

3D vector pMRE was successful in all participants. Two subjects, one with mild LUTS and one with severe LUTS, were excluded from the tissue stiffness analysis because pMRE was not performed at a frequency of 90 Hz. MRI and 3D vector pMRE at 90 Hz (19/21) data are summarized in Table 2. Men with moderate/severe LUTS had a higher prostate TZ volume (17 vs. 7 mL, *p* = 0.032) that men without LUTS. Prostate volume, PUL and BWT were similar across groups. Increase in PUL correlated with higher IPSS (ρ = 0.58; *p* = 0.023) and lower Qmax (ρ = −0.52; *p* = 0.026) (Fig. 2).

**Fig. 3:**
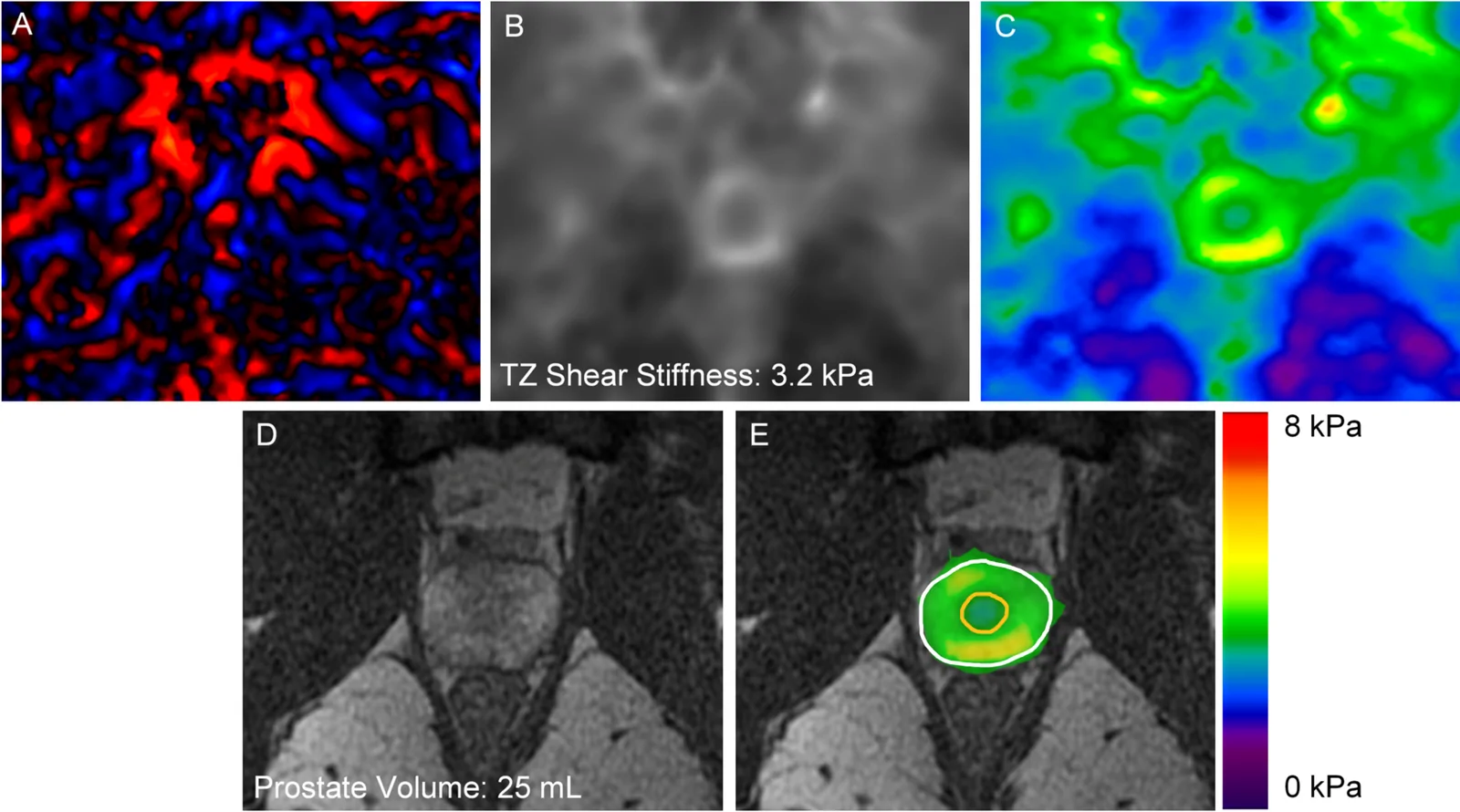
Magnetic resonance imaging (MRI) and 3D vector prostate magnetic resonance elastography (pMRE) of a 40-year-old man without benign prostate hyperplasia/lower urinary tract symptoms (BPH/LUTS). Axial wave image (**A**), shear stiffness (**B**) and color maps (**C**), T2-weighted (T2w) image (**D**) and fusion of shear stiffness color map on T2w image (**E**) of the prostate, at the midgland. On the fusion image, the prostate is outlined in white and the transition zone (TZ) in yellow. The mean prostate TZ shear stiffness was 3.2 kPa. Notice the stiffness of the TZ is lower than the peripheral zone in the asymptomatic man

**Fig. 4:**
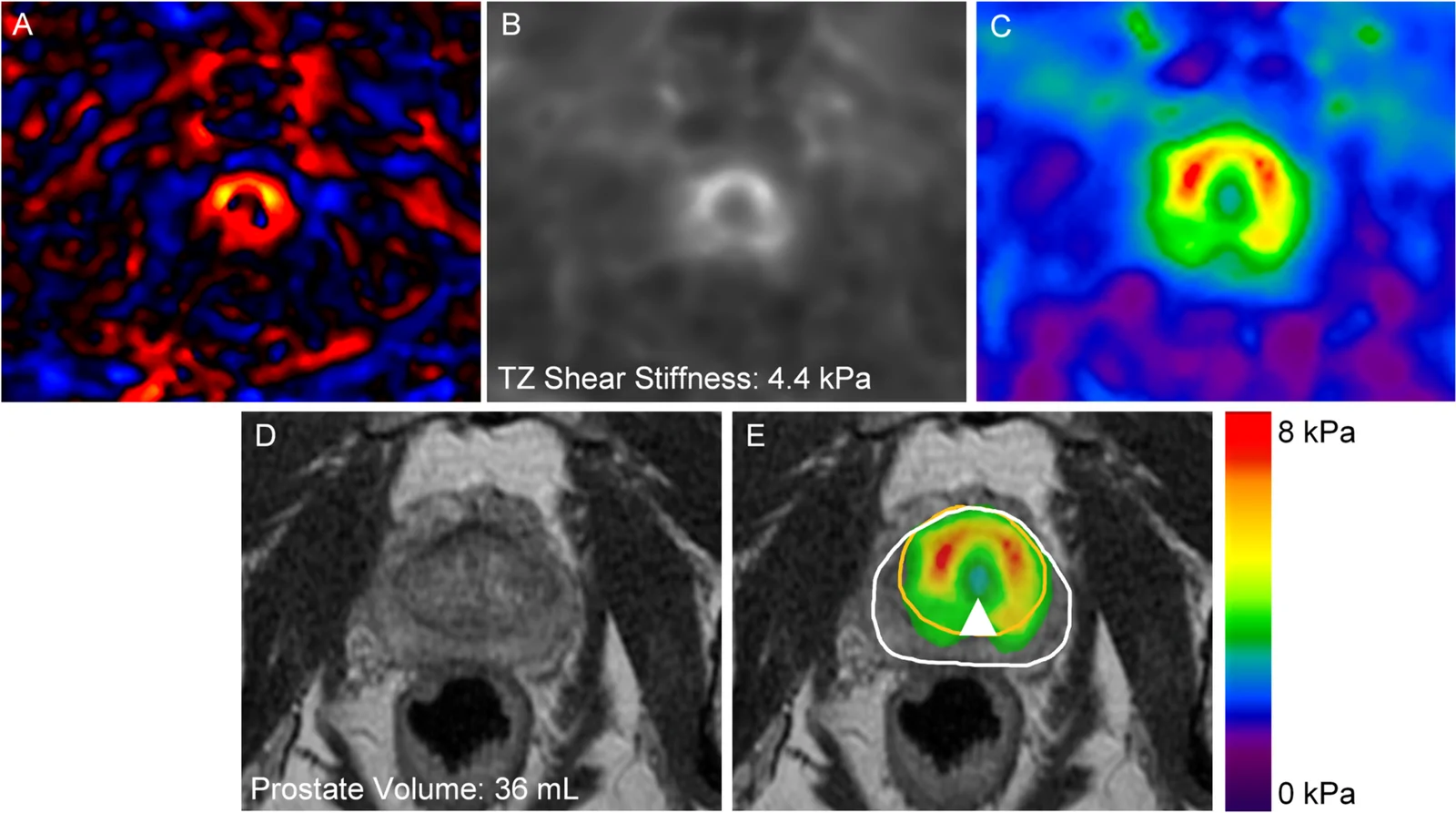
Magnetic resonance imaging (MRI) and 3D vector prostate magnetic resonance elastography (pMRE) of a 59-year-old man with moderate (IPSS = 15) benign prostate hyperplasia/lower urinary tract symptoms (BPH/LUTS). Axial wave image (**A**), shear stiffness (**B**) and color maps (**C**), T2-weighted (T2w) image (**D**) and fusion of shear stiffness color map on T2w image (**E**) of the prostate, at the midgland. On the fusion image, the prostate is outlined in white and the transition zone (TZ) in yellow. The mean prostate TZ shear stiffness was 4.4 kPa. Prostate and TZ volume are higher compared to the man without BPH/LUTS (Fig. 3) and there are heterogeneous stromal-predominant BPH-related changes throughout the TZ. Notice TZ shear stiffness heterogeneity with near-normal shear stiffness of the periurethral TZ (arrowhead). IPSS = International Prostate Symptom Score

Representative examples from prostate MRI and pMRE of a participant without and with moderate LUTS are displayed in Figs. 3 and 4, respectively. Higher midgland TZ shear stiffness was correlated with lower Qmax (ρ = −0.57; *p* = 0.017). Increase in the product of mean prostate TZ shear stiffness and PUL (kPa x U length) correlated with a lower Qmax.

(ρ = −0.59; *p* = 0.015) and Qave (ρ = −0.51; *p* = 0.04). The correlation between Qmax, Qave and voided volume was higher with [kPa x U length] compared to mean TZ shear stiffness alone. (Fig. 2). TZ shear stiffness was not correlated with prostate/TZ volume, PVR, or BWT.

### Tolerability

Individual responses to the tolerability questionnaire are summarized in Table 3. Most study participants found pMRE easy to tolerate without an urge to terminate the test prematurely.

**Table 3:** Magnetic resonance imaging (MRI) and 3D vector prostate magnetic resonance elastography (pMRE) tolerability questionnaire

	1 = Not at all	2 = A small amount	3 = A medium amount	4 = A large amount	Median (IQR)
It took a lot of effort to cope with the test?	17 (81%)	2 (9%)	2 (9%)	0	1 (1,1)
I experienced a metallic taste during my test?	16 (76%)	5 (24%)	0	0	1 (1,1.75)
I felt hot during my test?	9 (43%)	4 (19%)	7 (33%)	1 (5%)	2 (1,3)
I found the noise during my test easy to tolerate?	6 (29%)	4 (19%)	4 (19%)	7 (33%)	3 (1.25,4)
I felt calm during my test?	7 (33%)	1 (5%)	3 (14%)	10 (48%)	3.5 (1,4)
I wanted to come out of the machine during my test?	15 (71%)	3 (14%)	2 (10%)	1 (5%)	1 (1,2)
I felt a tingling sensation during my test?	9 (43%)	4 (19%)	5 (24%)	3 (14%)	2 (1,3)
I felt dizzy during my test?	20 (95%)	1 (5%)	0	0	1 (1,1)

A 4-point Likert scale was used to assess participant experience. Responses were counted and categorized according to score category and summarized with median (IQR)

## Discussion

In this prospective, cross-sectional study, we found that prostate magnetic resonance elastography with a novel, transperineal passive driver with an organ-specific mechanical frequency of 90 Hz is feasible and that higher prostate transition zone (TZ) shear stiffness and the product of TZ shear stiffness and prostate urethral length (PUL) were associated with voiding dysfunction, as measured by uroflowmetry. Specifically, we found that higher midgland TZ shear stiffness was associated with lower Qmax while longer PUL was associated with higher IPSS and lower Qmax. Higher mean TZ shear stiffness and PUL (kPa x U length) was associated with lower Qmax and Qave. Importantly, we found that the correlation between TZ shear stiffness, PUL and voiding parameters (Qmax and Qave) improved when TZ shear stiffness and urethral length were combined. These findings support our hypothesis that BPH-related changes in the prostate elongate and compress the urethra, limit urethral caliber during voiding and contribute to higher voiding pressure and lower flow rate, as defined by Hagen-Poiseuille’s Law.

While we saw a generally positive relationship between TZ shear stiffness and symptoms as measured by the IPSS, we did not find a statistically significant association with symptom scores across LUTS stratifications (none, mild, moderate-severe), likely due to lack of power in this feasibility study. It is important to recognize that symptom scores are subjective and other common causes of LUTS, including overactive bladder (OAB) and detrusor underactivity (DU), have similar and overlapping symptoms with BOO. Further, DU can lead to a pattern of voiding dysfunction similar to BOO, making it difficult to distinguish these conditions on uroflowmetry [5, 22–24]. Modeling the effect of TZ shear stiffness and prostatic urethra length in men against the bladder outlet obstruction index (BOOI) from pressure-flow studies (i.e. UDS) is the logical next phase in this line of research. We hypothesize that men with a BOOI > 40, indicative of bladder outlet obstruction, will have significantly higher TZ shear stiffness and combined TZ shear stiffness and urethral length compared to unobstructed men (BOOI < 20) [25–27].

Beyond excluding clinically significant prostate cancer, likely to change clinical management, employing mpMRI including pMRE in the evaluation of men with BPH/LUTS offers several advantages compared to existing diagnostic tests, such as ultrasound and UDS. MRI offers morphologic (i.e., intravesical protrusion) and volumetric assessment (PVR, PV, TZ volume, TZ index) of the prostate and bladder, that is highly accurate and reproducible [28]. The failure rate of pMRE is anticipated to be much lower than UDS. The pMRE failure rate in this study was 0% compared to a failure/non-diagnostic rate of 19–44% for UDS. [29, 30]. The cost of prostate MRI and pMRE compares favorably to UDS. Prostate MRI without contrast has an estimated technical fee of ~$230 and professional fee of ~$70 from the Centers of Medicare and Medicaid Services (CMS); the addition of MRE increases the fee by approximately $220 [31]. CMS reimbursement for UDS varies considerably based upon the facility code and the addition of concomitant procedures such as intrabdominal and urethral pressure profiles, electromyography studies, fluoroscopy, and uroflowmetry. In 2025, the global fee (technical + professional fee) for UDS performed for men with suspected BOO was ~$600 [32, 33]. Importantly, these costs do not include the charges associated with managing procedure-related complications associated with UDS including urinary tract infection, pain, or acute retention. Finally, patients tolerate MRI better than pressure-flow studies and are therefore more likely to complete the examination and obtain diagnostic results [34]. As a result, more men may be able to undergo comprehensive diagnostic evaluation prior to development of a treatment plan.

This prospective, cross-sectional study highlights the potential power of applying quantitative MRI techniques to men with LUTS but has several important limitations that require additional investigation. While the study poxation was sufficient to explore feasibility of the transperineal passive driver, most of the participants were not representative of clinical BPH/LUTS patients. Further, symptomatic men with voiding dysfunction in our cohort were not diagnosed with BOO. Given the overlap in symptoms and voiding dysfunction between BOO, OAB and DU, these conditions represent important unmeasured confounders. Future studies will incorporate UDS into the study design. While the mechanical frequency of 90 Hz provided sufficient image quality, the optimal frequency for pMRE remains unknown. Lastly, test-retest repeatability and reproducibility were beyond the scope of this study but are important to the validation of any image-based biomarker.

In conclusion, 3D vector prostate magnetic resonance elastography with a novel transperineal passive driver is feasible and can be used to measure prostate TZ shear stiffness. We found that higher TZ shear stiffness and longer PUL are associated with voiding dysfunction in men with moderate/severe LUTS. These findings suggest the potential of pMRE-derived TZ shear stiffness (and TZ shear stiffness x PUL) as non-invasive biomarkers, pending validation against pressure-flow studies.

## Data Availability

Data supporting the findings of this study are available within the manuscript. Additional data is available upon reasonable request from the corresponding author.
